# Self-managed weight loss by smart body fat scales ameliorates obesity-related body composition during the COVID-19 pandemic: A follow-up study in Chinese population

**DOI:** 10.3389/fendo.2022.996814

**Published:** 2022-11-09

**Authors:** Xinru Huang, Mingjie Li, Yefei Shi, Hongyun Yao, Zhijun Lei, Wenxin Kou, Bo Li, Jiayun Shi, Weiwei Zhang, Weixia Jian

**Affiliations:** ^1^ Department of Endocrinology, Xinhua Hospital, Shanghai Jiaotong University School of Medicine, Shanghai, China; ^2^ Department of Cardiology, Shanghai Tenth People’s Hospital, Tongji University School of Medicine, Shanghai, China

**Keywords:** COVID-19, weight loss, obesity, smart body fat scales, normal weight obesity

## Abstract

**Background:**

Since 2020, longer stay-at-home time in response to the coronavirus disease 2019 (COVID-19) pandemic has changed the weight-related behaviors of Chinese population.

**Objectives:**

To explore the demographic and basic characteristics of body fat scale users and to investigate the changes in obesity-related body composition of overweight and obese users during COVID-19. Further, we analyzed the factors associated with successful weight loss and improved body composition changes in overweight and obese people.

**Methods:**

The study included 107,419 Chinese adults registered in the smart app connecting to the body fat scale in 2020 to describe the demographic characteristics of body fat scale users by Unpaired Student’s t-test and Chi-Square test. Subsequently, overweight and obese participants with body mass index (BMI) of more than 24 kg/m^2^ were screened to investigate the independent factors associated with effective weight loss and improved body composition changes by multivariable logistic regression analyses.

**Results:**

During the pandemic, the number of body fat scale users increased markedly compared with pre-pandemic. Over half of the participants were women and with normal baseline BMI. Based on BMI classification, multivariable logistic regressions showed that age, gender, measurement frequency classification, baseline BMI, visceral adipose index and skeletal muscle rate were associated with weight loss and fat loss in the overweight and obese population, with the high-frequency measurement being the most important factor for effective weight and fat loss. In the population with normal BMI obesity, younger age was the most significant factor for effective fat loss.

**Conclusion:**

During the COVID-19 pandemic, participation in self-monitored weight loss increased markedly compared with pre-pandemic, and women accounted for the majority. We found that many overweight and obese participants achieved weight loss goals by smart body fat scales, and the effectiveness of weight and fat loss was greater in obese participants than in overweight participants, both based on BMI and PBF classification. In addition, promoting the usage of smart body fat scales could contribute to more effective weight and fat loss in the overweight and obese population based on BMI classification. However, in the population with normal BMI obesity, young subjects might be easier to successfully lose fat compared with the elder. Digital self-management by smart body fat scales could become a promising approach for the obese population with high BMI to lose weight and keep healthy.

## Introduction

Due to the rapid improvement of economic level and the westernization of dietary habits in China, the proportion of overweight and obese patients in the adult population climbed from 18.9% and 2.9% in 2002 to 34.3% and 16.4% in 2020, respectively (1). According to previous studies, obesity and metabolic syndrome were closely related to the occurrence and development of diabetes (2), hyperlipidemia, cardiovascular disease (3), cerebrovascular disease (4), and cancers (5). Obesity has become a critical metabolic disease threatening people’s health and is the most common comorbidity reported among patients with severe coronavirus disease 2019 (COVID-19) (6), thus the prevention and treatment of obesity care are crucial to avoid complications and a high rate of hospitalization. Several studies confirmed the potential efficacy of lifestyle interventions in reducing obesity in Caucasians (7, 8). These studies found that interventions, such as establishing appropriate weight loss goals, self-monitoring, and adjusting dietary intake and physical activity, could achieve the purpose of weight loss (9). Because of stay-at-home for more time than before, Chinese people have changed their daily behaviors including dietary intake and physical activity during the COVID-19 pandemic (10).

On account of the progression of the Internet and smartphones, various types of online health management software have appeared. Wang Y analyzed 17 reviews and found that health self-management depending on mobile apps was beneficial to the treatment of diabetes and obesity (11). In the meta-analysis by Flores M et al., patients using online methods had significantly greater reductions in body weight and body mass index (BMI) compared with patients using other traditional methods such as diary records (12). Notably, the frequency of self-weighing was higher in the group that uploaded weight data *via* Bluetooth compared with the group that entered weight data manually, and the percentage of participants who achieved weight-loss goals was also noticeably higher (13).

BMI is the most commonly used indicator for diagnosing obesity. At present, 24 kg/m^2^ and 28 kg/m^2^ of BMI values are used as the boundary values for being overweight and obese in China (14). However, it fails to determine whether the changes in BMI caused by fat mass (FM) or non-fat mass (FFM) (15). The concept of percentage of body fat (PBF) refers to the proportion of body fat mass in total body weight. Normal BMI obesity refers to people with normal BMI and high PBF. Previous studies found this specific population was susceptible to metabolic disorders and PBF could be used to help assess the metabolic risk (16–18). Thus, it is more reliable to establish PBF as an index to assess the improvement effect of body composition in this specific population. Nevertheless, most previous studies on the effect of weight loss were carried out in European Caucasians. Few studies focused on the effectiveness of self-managed weight loss *via* body fat scales in Chinese population, especially during the COVID-19 pandemic.

According to the aforementioned studies (11–13), online software-based self-management helped weight loss and alleviated diabetes and obesity. However, the effects of self-management based on online software and its independent factors during the COVID-19 pandemic are unclear, which are our current study concerns. Here, we conducted a cohort study based on the data of obesity-related anthropometric indices from the Qingniu Health app connecting to the body fat scale in overweight and obese people for at least one year self-monitoring, to test our hypotheses that online software might help self-management during the COVID-19 pandemic and measurement frequency would be an important factor.

## Materials and methods

### Subjects

**Figure 1:**
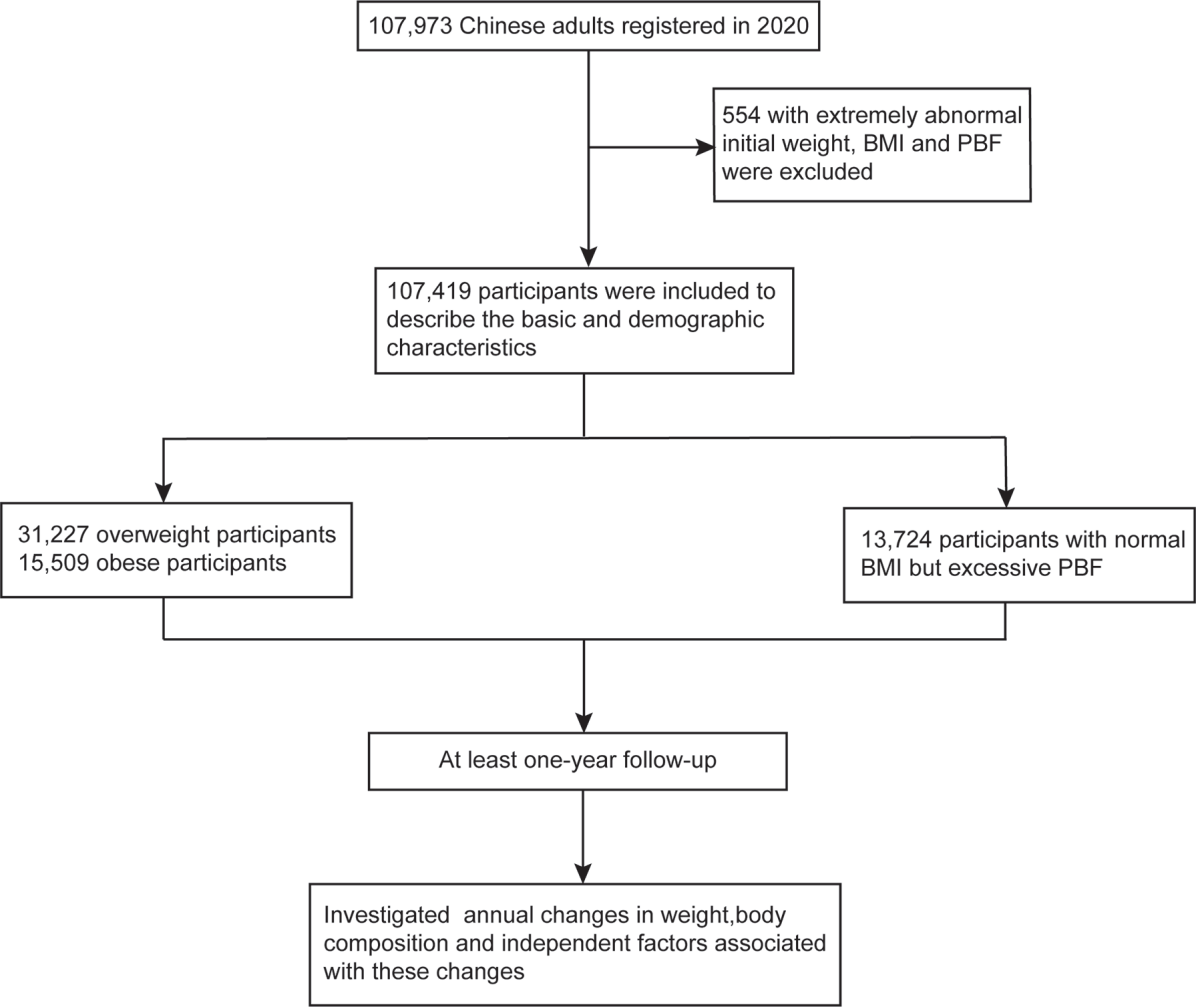
Flowchart of participant inclusion. BMI, Body Mass Index; PBF, Percentage of Body Fat.

The inclusion of participants were adult users aged 18 to 79 years in China, who signed up to use the Qingniu Health app connecting to the body fat scale in 2020 during the COVID-19 pandemic. Participants with extremely abnormal initial weight (below 30kg), BMI (below 14 kg/m^2^ and above 60 kg/m^2^) and PBF (below 10%) were excluded. Furthermore, participants whose annual measurement frequency was less than 2 times were also excluded. 107,419 subjects were included to describe the basic and demographic characteristics of body fat scale users registered in 2020. Then, 31,227 overweight participants with BMI of 24-27.9 kg/m^2^ and 15,509 obese participants with BMI ≥28 kg/m^2^ were followed up to investigate the improvement of obesity-related body composition indices for at least one year, which meant the time span from the first measurement to the last measurement was more than 1 year. Moreover, 13,724 participants with normal BMI but excessive PBF were also followed up (**Figure 1**). Electronic consent for the inclusion of participants was obtained. The study was approved by Xinhua Hospitals’ Ethics Committee.

### Data collection

Data were collected in the Qingniu Health app, which contained the baseline information of participants at the time of initial registration, including gender, age, height, and residential city, as well as the measurements including weight, PBF, visceral adipose index (VAI), skeletal muscle rate (SMR) and fat-to-muscle ratio (FMR) by using the same brand, the same type of body fat scales (CS10C; Yolanda Technology Co., Ltd., Shenzhen, China). The measurement principle of the body fat scales is the multi-frequency bioelectrical impedance analysis (BIA), as same as the previous study (19). The participant’s body fat scales measurement data were synchronized to the Qingniu Health app platform *via* Bluetooth.

In the following analyses, participants were grouped by gender, age, BMI, PBF, measurement frequency, and the residential city level, respectively. Participants aged <40, 40 - 59, and ≥60 years were defined as the adults, middle-aged and elderly group. Participants of BMI <18.5 kg/m^2^, 18.5 - 23.9 kg/m^2^, 24 - 27.9 kg/m^2^, and ≥28 kg/m^2^ were considered as underweight, normal, overweight and obesity respectively. Male with PBF ≥25.0% and female with PBF ≥30.0% were regarded as obese (20, 21), and the others were regarded as non-obese subjects. Normal BMI obesity refers to people with normal BMI and high PBF. Participants were categorized into tertiles of measurement frequency as low, middle, and high-frequency groups. As the usage of body fat scales was irregular during the follow-up period, we adopted the last measurement indices at least more than 1 year from baseline as the end-point results. The annual changes in weight, PBF and SMR were calculated as follows: (last measurement value – initial measurement value)/time (month) * 12. A study showed weight loss by 5% of initial weight reduced the risk of obesity-related comorbidities (22). Therefore, we defined participants with a decrease of more than 5% of initial body weight as effective weight loss. Likewise, a decrease of more than 5% of initial PBF was considered effective fat loss (23). The annual growth rate of SMR increased by more than 5%, which was also considered beneficial muscle gain.

### Statistical analyses

Continuous variables were described as mean and standard deviation (SD), measurement frequency was described as median and interquartile range (IQR), and categorical variables were described as count and percentage. Unpaired Student’s t-test or one-way ANOVA was used to analyze differences among groups, and differences in constituent ratios were analyzed by Chi-Square test. Univariable and multivariable logistic regression analyses were used to assess the independent influencing factors of the annual change rates of body weight, PBF, and SMR, respectively. Statistical analyses were performed using the Statistical Package for the Social Sciences (SPSS v.26.0, SPSS Inc.). Statistically significance was set at p value <0.05.

## Results

### General baseline characteristics of body fat scale users during the COVID-19 pandemic

The annual number of users registered in 2020 during the pandemic (N=107,419) was significantly higher than that in 2019 before the pandemic (N=73,307). During the pandemic, over half of the participants were women (84.7%) and with normal BMI. On average, participants were 32.87 ± 8.51 years and consisted of underweight (4.1%), normal (52.4%), overweight (29.1%), and obesity (14.4%) (**Table 1**). Participants in economically developed cities such as first-tier, new first-tier, and second-tier cities accounted for 86.5%. According to the users’ annual measurement frequency, participants were divided into three groups: low, middle, and high-frequency groups (13.33 IQR: 10.53; 42.03 IQR: 21.58; and 129.73 IQR: 101.61 times per year). At baseline, the average age of female users was younger than that of males (P <0.0001); the average weight of men was 79.13 ± 13.97 kg, and that of women was 61.62 ± 11.12 kg (P <0.0001); the average BMI of men was significantly higher than that of women (26.26 ± 4.20 vs 23.59 ± 4.00 kg/m^2^, P <0.0001), the proportion of overweight and obese users was also higher in men than in women (69.4% vs 38.9%, P <0.0001). Interestingly, the largest proportion of female users was in the normal BMI group (56.4%), and the average annual measurement frequency of female users was much higher than that of male users (44.44 IQR: 79.62 vs 30.89 IQR: 52.45 times per year, P <0.0001).

**Table 1:** Baseline characteristics of the participants grouped by gender during the COVID-19 pandemic.

	Total	Man	Woman	p-Value*
N (%)	107419 (100)	16447 (15.3)	90972 (84.7)	/
Age (years)	32.87 (8.51)	33.25 (9.95)	32.80 (8.22)	<0.0001
Classification (Age, years)		N (% of N_column_)		
[18, 40)	87197 (81.2)	12731 (77.4)	74466 (81.9)	<0.0001
[40, 60)	19375 (18.0)	3414 (20.8)	15961 (17.5)	
[60, 80)	847 (0.8)	302 (1.8)	545 (0.6)	
Baseline weight (kg)	64.30 (13.20)	79.13 (13.97)	61.62 (11.12)	<0.0001
Baseline BMI (kg/m^2^)	24.00 (4.14)	26.26 (4.20)	23.59 (4.00)	<0.0001
Classification (BMI, kg/m^2^)		N (% of N_column_)		
Underweight (<18.5)	4392 (4.1)	94 (0.6)	4298 (4.7)	<0.0001
Normal [18.5~24)	56291 (52.4)	4940 (30.0)	51351 (56.4)	
Overweight [24~28)	31227 (29.1)	6781 (41.2)	24446 (26.9)	
Obesity [>28)	15509 (14.4)	4632 (28.2)	10877 (12.0)	
Baseline PBF (%)	29.50 (5.50)	25.08 (5.75)	30.30 (5.06)	<0.0001
Classification (PBF, %)		N (% of N_column_)		
Normal (<25/30)	50022 (46.6)	7806 (47.5)	42216 (46.4)	0.012
Obesity [>25/30)	57397 (53.4)	8641 (52.5)	48756 (53.6)	
Baseline SMR (%) Baseline FMR	41.72 (4.00) 0.72 (0.20)	47.79 (3.57) 0.54 (0.17)	40.62 (2.95) 0.76 (0.18)	<0.0001 <0.0001
Baseline VAI	6.58 (3.76)	8.73 (3.86)	6.19 (3.61)	<0.0001
Measurement frequency (times/year)	42.03 (75.47)	30.89 (52.45)	44.44 (79.62)	<0.0001
Classification (Frequency)		N (% of N_column_)		
Low	35812 (33.3)	7022 (42.7)	28790 (31.6)	<0.0001
Middle	35795 (33.3)	5563 (33.8)	30232 (33.2)	
High	35812 (33.3)	3862 (23.5)	31950 (35.1)	
Classification (City)		N (% of N_column_)		
First-tier	24482 (33.4)	4138 (36.6)	20344 (32.8)	<0.0001
New first-tier	24420 (33.3)	3759 (33.2)	20661 (33.3)	
Second-tier	14541 (19.8)	2148 (19.0)	12393 (20.0)	
Third-tier	5884 (8.0)	761 (6.7)	5123 (8.3)	
Fourth-tier	3066 (4.2)	383 (3.4)	2683 (4.3)	
Fifth-tier	987 (1.3)	122 (1.1)	865 (1.4)	

Continuous variables were described as mean (SD), “Measurement frequency” was described as median (IQR), and categorical variables were described as count (percentage). *All p values were compared between different gender. BMI, Body Mass Index; PBF, Percentage of Body Fat; SMR, Skeletal Muscle Rate; FMR, Fat-to-Muscle Ratio; VAI, Visceral Adipose Index.

### Annual changes in weight and body composition for overweight and obese participants based on BMI classification

**Figure 2:**
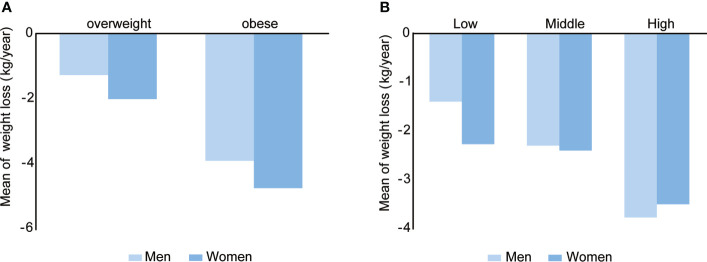
Mean of weight loss during the COVID-19 period in different BMI **(A)** and measurement frequency **(B)** groups. Overweight, men: n=6781, women: n=24446; obese, men: n=4632, women: n=10877. Low frequency, men: n=4475, women: n=8602; middle frequency, men: n=3900, women: n=11117; High frequency, men: n=3038, women: n=15604.

A total of 46,736 overweight and obese participants (N=31,227, 15,509 respectively) at baseline who had healthy needs of weight loss were followed up for longer than one year. There was no statistical difference in the annual weight loss of participants among different age groups. Nevertheless, the group aged 18-40 years experienced a greater decrease in PBF and a greater increase in SMR compared with the group aged 40-60 years. According to BMI classification, obese participants showed greater changes in all indices, including weight, BMI and PBF compared with overweight participants (P <0.0001). In obese men, on average, the annual body weight decreased by 3.90 ± 7.77 kg (**Figure 2A**), and the BMI decreased by 1.42 ± 2.59 kg/m^2^. In obese women, the annual weight loss was 4.74 ± 8.78 kg, and the BMI decreased by 1.80 ± 3.36 kg/m^2^. Grouped by PBF, obese participants showed greater changes in all indices than normal individuals as well (P <0.0001). Among different measurement frequency groups, the changes of all obesity-related indices in the high-frequency group were significantly greater than those of the low and middle-frequency groups (P <0.0001) (**Figure 2B**; **Table 2**).

**Table 2:** Changes of obesity-associated body composition associated with baseline characteristics.

	Weight (kg)	BMI (kg/m^2^)	PBF (%)	SMR (%)
Age (years)				
Man				
18-40	-2.34 (6.36)	-0.86 (2.13)	-0.99 (2.78)*	0.64 (1.83)*
40-60	-2.25 (4.64)	-0.80 (1.57)	-0.82 (2.11)*	0.54 (1.43)*
60-80	-2.55 (4.26)	-0.90 (1.38)	-0.99 (1.99)	0.67 (1.36)
*p-*Value	0.566	0.226	0.007	0.017
Woman				
18-40	-2.88 (6.26)*	-1.08 (2.40)	-1.03 (2.30)*	0.60 (1.35)*
40-60	-2.68 (4.98)*	-1.02 (1.95)	-0.82 (1.79)*	0.48 (1.06)*
60-80	-2.87 (5.42)	-1.11 (2.12)	-0.84 (1.79)	0.50 (1.05)
*p-*Value	0.016	0.102	<0.0001	<0.0001
BMI (kg/m^2^)				
Man				
24-28	-1.26 (4.08)	-0.46 (1.36)	-0.59 (2.21)	0.39 (1.49)
≥28	-3.90 (7.77)	-1.42 (2.59)	-1.48 (3.09)	0.96 (2.02)
*p-*Value	<0.0001	<0.0001	<0.0001	<0.0001
Woman				
24-28	-2.00 (3.97)	-0.75 (1.55)	-0.78 (1.89)	0.46 (1.11)
≥28	-4.74 (8.78)	-1.80 (3.36)	-1.45 (2.73)	0.85 (1.60)
*p-*Value	<0.0001	<0.0001	<0.0001	<0.0001
PBF (%)				
Man				
<25	-0.80 (3.88)	-0.31 (1.26)	-0.22 (2.23)	0.18 (1.48)
≥25	-2.83 (6.48)	-1.02 (2.18)	-1.19 (2.72)	0.77 (1.81)
*p-*Value	<0.0001	<0.0001	<0.0001	<0.0001
Woman				
<30	-1.46 (3.88)	-0.47 (1.37)	0.59 (2.89)	-0.40 (1.77)
≥30	-2.85 (6.03)	-1.07 (2.32)	-1.00 (2.20)	0.58 (1.29)
*p-*Value	<0.0001	<0.0001	<0.0001	<0.0001
Measurement frequency classification				
Man				
Low	-1.39 (6.29)	-0.52 (2.07)	-0.60 (2.80)	0.39 (1.87)
Middle	-2.29 (5.63)	-0.85 (1.91)	-0.95 (2.55)	0.63 (1.65)
High	-3.76 (5.76)	-1.32 (1.97)	-1.48 (2.42)	0.94 (1.63)
*p-*Value	<0.0001	<0.0001	<0.0001	<0.0001
Woman				
Low	-2.26 (6.98)	-0.84 (2.67)	-0.77 (2.47)	0.45 (1.46)
Middle	-2.39 (5.94)	-0.90 (2.28)	-0.83 (2.17)	0.48 (1.27)
High	-3.49 (5.42)*	-1.32 (2.10)*	-1.22 (2.04)*	0.71 (1.19)*
*p-*Value	<0.0001	<0.0001	<0.0001	<0.0001

All data were annual changing amplitude of obesity-associated body indices and expressed as mean (SD). * indicated that there were statistical differences among different groups.

### Independent factors associated with weight loss, fat loss, and muscle gain

**Figure 3:**
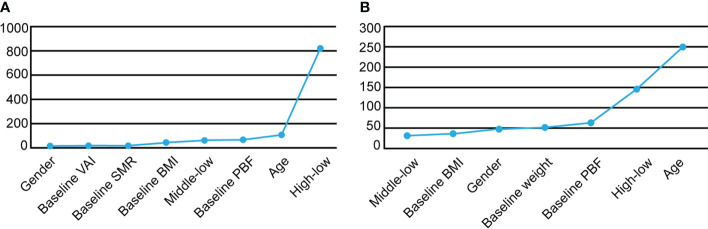
The importance of factors in logistic regression model which was measured as the partial chi-square statistic minus the predictor degrees of freedom (χ^2^ - df) was shown. **(A)** χ^2^ - df values for the prediction of weight loss success in the overweight and obese population with BMI ≥24. **(B)** χ^2^ - df values for the prediction of effective fat loss in the obese population with normal BMI.

Based on the above findings, we further performed univariable and multivariable logistic regression analyses to find the independent factors associated with successful weight, fat loss and muscle gain. Univariable logistic analysis showed that age, gender, measurement frequency classification, baseline weight, BMI, PBF, VAI and SMR were associated with weight loss (**Table S1**). Multivariable logistic regression models revealed that participants of the female sex, with lower age, lower VAI, higher baseline BMI, PBF, SMR and measurement frequency were more likely to succeed in losing weight (**Table 3**). As described in Bajaj NS’ study (24), the importance of each factor in the logistic regression model was measured as the partial chi-square statistic minus the predictor degrees of freedom (χ^2^ - df). High measurement frequency was the largest predictor for effective weight loss (OR = 2.101, 95% CI 1.997-2.211, χ^2^ - df = 818.81, *P <*0.0001) when other associated factors were corrected, including age, gender, baseline BMI, PBF, VAI and SMR (**Figure 3A**).

**Table 3:** Factors associated with the effective change of weight and obesity-associated body composition.

Factors	Weight loss		Fat loss		Muscle gain	
	OR (95% CI)	*p-*Value	OR (95% CI)	*p-*Value	OR (95% CI)	*p-*Value
Age	0.985 (0.983-0.988)	<0.0001	0.970 (0.967-0.973)	<0.0001	0.958 (0.954-0.962)	<0.0001
Gender	1.461 (1.178-1.811)	0.001	0.318 (0.261-0.389)	<0.0001	0.112 (0.086-0.145)	<0.0001
Baseline BMI	1.129 (1.087-1.172)	<0.0001	1.161 (1.118-1.206)	<0.0001	1.209 (1.149-1.273)	<0.0001
Baseline PBF	1.082 (1.061-1.103)	<0.0001	/	/	/	/
Baseline VAI	0.916 (0.876-0.957)	<0.0001	0.836 (0.799-0.875)	<0.0001	0.798 (0.750-0.849)	<0.0001
Baseline SMR	1.066 (1.032-1.100)	<0.0001	0.913 (0.893-0.934)	<0.0001	0.750 (0.727-0.774)	<0.0001
Measurement frequency classification						
Middle-low	1.237 (1.172-1.305)	<0.0001	1.256 (1.188-1.329)	<0.0001	1.081 (0.994-1.177)	0.070
High-low	2.101 (1.997-2.211)	<0.0001	1.999 (1.895-2.108)	<0.0001	1.599 (1.479-1.729)	<0.0001

All p values were results of multivariable logistic regression analysis in participants with BMI ≥ 24 kg/m^2^.

Multivariable logistic regression analysis showed that lower age, male sex, lower baseline VAI, SMR, higher baseline BMI and measurement frequency were independent factors for effective fat loss (**Table 3**). Especially, high measurement frequency was a most contributing factor for success of fat loss (OR = 1.999, 95% CI 1.895-2.108, χ^2^ - df = 645.50, *P <*0.0001).

**Table S1** showed age, gender, measurement frequency classification, baseline weight, BMI, PBF, VAI and SMR were associated with muscle gain. Multivariable logistic regression models showed age was a most significant factor for muscle gain (OR = 0.958, 95% CI 0.954-0.962, χ^2^ - df = 384.43, P <0.0001) when other factors were corrected. Other than lower age, significant factors associated with successful muscle gain were male sex, higher measurement frequency, higher baseline BMI, lower baseline VAI and SMR (**Table 3**).

### Independent factors associated with fat loss in the population with normal BMI but excessive PBF

13,724 participants with normal BMI but excessive PBF were observed, and women accounted for 99.5% of this specific obese population. In the group with normal BMI, the measurement frequency was higher in people with excessive PBF than in people with normal PBF (51.51 IQR: 87.12 vs 33.87 IQR: 57.79, P <0.0001), which indicated that participants were not only concerned about changes in weight and BMI, but PBF management was also an important part of health monitoring (**Table S2**). Multivariable logistic regression analysis displayed male sex, lower age and BMI, higher baseline weight, PBF and measurement frequency were promoting factors for effective fat loss and muscle gain, among which, lower age was the most important contributing factor (**Table 4**; **Figure 3B**).

**Table 4:** Factors associated with the effective fat loss and muscle gain in participants with normal BMI but high PBF.

Factors	Fat loss		Muscle gain	
	OR (95% CI)	*p-*Value	OR (95% CI)	*p-*Value
Age	0.926 (0.918-0.935)	<0.0001	0.893 (0.879-0.907)	<0.0001
Gender	0.059 (0.027-0.130)	<0.0001	0.004 (0.002-0.012)	<0.0001
Baseline weight	1.056 (1.040-1.072)	<0.0001	1.067 (1.038-1.097)	<0.0001
Baseline BMI	0.688 (0.610-0.776)	<0.0001	0.631 (0.518-0.768)	<0.0001
Baseline PBF	1.314 (1.229-1.404)	<0.0001	1.495 (1.368-1.635)	<0.0001
Measurement frequency classification				
Middle-low	1.441 (1.272-1.632)	<0.0001	1.084 (0.819-1.436)	0.572
High-low	2.071 (1.841-2.330)	<0.0001	1.804 (1.399-2.327)	<0.0001

## Discussion

Obesity, closely related to chronic metabolic diseases such as hypertension and diabetes, endangers the health of Chinese (25, 26). Recently, the harm of obesity has become more visible, and people have paid more attention to weight management (27). More and more body fat scales accompanied with their supporting weight management apps have appeared, and have been popularized in Chinese population because of convenience and economy (28). However, the role of self-managed weight loss through the use of smart body fat scales in Chinese during the COVID-19 pandemic is to be assessed. Our present study is a follow-up study based on the data of a Chinese commercial online weight management app connecting a smart body fat scale. We found online software might help self-management during the COVID-19 pandemic and measurement frequency would be an important factor.

The COVID-19 pandemic has resulted in overall changes to weight-related behaviors, including dietary intake and physical activity (29). Based on the baseline characteristics of all included users, we found that the young and middle-aged women were the main population using body fat scales during the COVID-19 pandemic, which suggested that they were more concerned about weight, body composition, and their health conditions compared with other groups. Furthermore, female users with normal BMI were more than half, which suggested that women placed more emphasis on overweight and obesity prevention than men, consistent with other research findings (28). Overweight persons predominated in male participants, nevertheless, their annual average measurement frequency was lower than female users. Thus, it was necessary to increase men’s awareness of preventing obesity in daily life through self-managed weight loss. In addition, participants in economically developed cities accounted for 86.5%, reflecting that the degree of urban development and economic level affected the usage of smart body fat scales.

Self-monitoring was the core of behavioral intervention for weight loss, including weight monitoring. Several studies found that self-monitoring was positively correlated with weight loss (30–33). However, the previous researches had some limitations, for example, the subjects of those study were a relative small sample size and confined to Caucasians. In the present study, we found the changes in weight, BMI and obesity-associated body composition were significantly different among different age, baseline BMI, PBF and measurement frequency groups in the overweight and obese participants. The changes in body composition were more obvious in the young, compared with the middle-aged person. The annual average weight loss of obese men was 3.90kg and that of obese women was 4.74kg, which implied that many overweight and obese participants based on BMI classification still achieved weight loss goals during the COVID-19 epidemic. According to PBF classification, the annual average weight loss of obese men was 2.83kg and that of obese women was 2.85kg, which showed a greater reduction than participants with normal PBF. At the same time, we found that the values of weight loss and fat loss were higher in obese participants than in overweight participants, both based on BMI and PBF classification, which indicated high PBF was also an important index, as in other studies (34). The current study provided evidence for the value of self-managed weight loss by using an online weight management app in the overweight and obese population.

A clinical study published by Carter et al. found that the participant’s compliance with weight monitoring by smartphone was significantly improved compared with paper diary and website monitoring (35). In Thomas’s study, participants with a BMI of 27-40 kg/m^2^ weighed using a commercial online smart scale and received active weight loss therapy simultaneously. This study showed that higher weight monitoring frequency was associated with better weight loss, which was consistent with numerous studies (13, 33). Bennett and colleagues found that the weight loss of participants with more frequent self-weighing was significantly higher than others in the 12-month digital obesity treatment (36). The sustainability of long-term self-monitoring was a strong predictor of weight loss success through lifestyle interventions (37), which was confirmed by our study that the high-frequency monitoring group had greater weight loss, fat loss, and muscle gain in the overweight and obese population classified by BMI. For successful weight loss and fat loss, high-frequency measurement was the most significant promoting factor in our study, while some other related factors were not included, like lifestyle changes. However, younger age was the most significant factor for effective muscle gain when other factors were corrected, it meant younger people gained muscle more easily than older people. Interestingly, we found that younger age was the most contributing factor for effective fat loss in the population with normal BMI obesity, and moderate self-management by using a smart body fat scale could contribute to successful fat loss and muscle gain in the young people, which might be related to higher metabolic rates in young people (38). This interesting finding from our study suggests that early weight-related intervention at a younger age may lead to more successful fat loss in normal BMI obese people.

Our study revealed the beneficial role of self-monitoring of weight and body composition by a smart body fat scale in self-managed weight loss and amelioration of body composition in Chinese overweight and obese people during the COVID-19 pandemic. Moreover, we indicated promoting the usage of smart body fat scales could contribute to weight and fat loss in the overweight and obese population with high BMI, thus promoting remote weight self-monitoring by smart body fat scales in clinical practice might help the progression of weight loss in obese patients. However, there were also some limitations in our study, one of which was that participants’ conditions were not taken into account, such as diabetes and thyroid-related disease, which might affect body composition. Moreover, other contributing factors of weight loss, such as dietary intake or physical activity were not included during the follow-up periods, and some accidental conditions might affect the accuracy of body fat measurements by BIA in our real-world study, such as the hydration status. A large randomized intervention trial could be performed to reinforce the results of this study. Nevertheless, this study analyzed a sizable population from the majority of Chinese urban cities, which implied the potential advantages of health economics through self-monitored weight loss by smart body fat scales.

In conclusion, during the COVID-19 pandemic, participation in self-monitored weight loss increased markedly compared with pre-pandemic, and women accounted for the majority. We found that many overweight and obese participants achieved weight loss goals by smart body fat scales, and the effectiveness of weight and fat loss was greater in obese participants than in overweight participants, both based on BMI and PBF classification. In addition, promoting the usage of smart body fat scales could contribute to more effective weight and fat loss in the overweight and obese population by BMI classification. However, in the population with normal BMI obesity, young subjects might be easier to successfully lose fat compared with the elder. Digital self-management by smart body fat scales could become a promising approach for the obese population with high BMI to lose weight and keep healthy.

## Data availability statement

The datasets presented in this article are not readily available because restrictions apply to the availability of these data. Data were obtained from the Qingniu Health app and are available from the authors with the permission of the Qingniu Health app. Requests to access the datasets should be directed to Weixia Jian, jianweixia@xinhuamed.com.cn.

## Ethics statement

The studies involving human participants were reviewed and approved by Xinhua Hospitals’ Ethics Committee. Written informed consent for participation was not required for this study in accordance with the national legislation and the institutional requirements.

## Author contributions

XH, ML, YS, HY, ZL, WK, BL, JS, WZ and WJ implemented the study. XH, ML, and WJ wrote the manuscript. YS, HY and ZL analyzed the data. All authors participated in the design of the studies, analysis of the data and review of the manuscript. All authors contributed to the article and approved the submitted version.

## Funding

This research was funded by the Natural Science Foundation of Shanghai, grant number 20ZR1435300.

## Acknowledgments

We would like to thank all participants in this study and the Qingniu Health app.

## Conflict of interest

The authors declare that the research was conducted in the absence of any commercial or financial relationships that could be construed as a potential conflict of interest.

## Publisher’s note

All claims expressed in this article are solely those of the authors and do not necessarily represent those of their affiliated organizations, or those of the publisher, the editors and the reviewers. Any product that may be evaluated in this article, or claim that may be made by its manufacturer, is not guaranteed or endorsed by the publisher.
