# Supplementary material for: Self-managed weight loss by smart body fat scales ameliorates obesity-related body composition during the COVID-19 pandemic: A follow-up study in Chinese population
Source: Front Endocrinol (Lausanne). 2022 Nov 9;13:996814. doi: 10.3389/fendo.2022.996814 (PMC9682041; doi:10.3389/fendo.2022.996814)
Supplement: Supplementary file 1 [file DataSheet_1.docx]

**Self-Managed Weight Loss by Smart Body Fat Scales Ameliorates Obesity-Related Body Composition** **during the COVID-19 Pandemic: a Follow-Up Study in Chinese Population**

**Xinru Huang^1^†, Mingjie Li^1^†, Yefei Shi^2^, Hongyun Yao^1^, Zhijun Lei^2^, Wenxin Kou^2^, Bo Li^2^, Jiayun Shi^2^, Weiwei Zhang^1^, Weixia Jian^1*^**

^1^Department of Endocrinology, Xinhua Hospital, Shanghai Jiaotong University School of Medicine, Shanghai, China.

^2^Department of Cardiology, Shanghai Tenth People’s Hospital, Tongji University School of Medicine, Shanghai, China.

*** Correspondence:**

Weixia Jian, jianweixia@xinhuamed.com.cn.

Xinru Huang^1^† and Mingjie Li^1^†. These authors contributed equally to this work and share first authorship.

**Table S1.** Factors associated with the effective change of weight and body composition by univariate logistic regression analysis

| **Factors** | **Weight loss** | | **Fat loss** | | **Muscle gain** | |
| --- | --- | --- | --- | --- | --- | --- |
|  | OR (95% CI) | *p-*Value | OR (95% CI) | *p-*Value | OR (95% CI) | *p-*Value |
| Age | 0.991  (0.989-0.993) | <0.0001 | 0.977  (0.974-0.979) | <0.0001 | 0.972  (0.968-0.976) | <0.0001 |
| Gender | 1.649  (1.573-1.730) | <0.0001 | 0.779  (0.744-0.816) | <0.0001 | 1.170  (1.090-1.256) | <0.0001 |
| Frequency classification | 1.499  (1.463-1.536) | <0.0001 | 1.338  (1.304-1.372) | <0.0001 | 1.266  (1.220-1.314) | <0.0001 |
| Baseline weight | 1.011  (1.009-1.013) | <0.0001 | 1.014  (1.013-1.016) | <0.0001 | 1.030  (1.028-1.033) | <0.0001 |
| Baseline BMI | 1.077  (1.071-1.083) | <0.0001 | 1.047  (1.041-1.052) | <0.0001 | 1.131  (1.124-1.139) | <0.0001 |
| Baseline PBF  Baseline VAI  Baseline SMR | 1.079  (1.074-1.084)  1.084  (1.078-1.091)  0.925  (0.920-0.930) | <0.0001  <0.0001  <0.0001 | 1.004  (1.000-1.009)  1.050  (1.044-1.057)  1.008  (1.003-1.013) | 0.054  <0.0001  0.001 | 1.108  (1.100-1.116)  1.149  (1.140-1.158)  0.907  (0.899-0.915) | <0.0001  <0.0001  <0.0001 |
| City classification | 0.991  (0.972-1.010) | 0.335 | 0.969  (0.949-0.989) | 0.002 | 0.973  (0.944-1.002) | 0.067 |

**Table S2.** Characteristics of participants with normal BMI but excessive PBF

| **Variables** |  |
| --- | --- |
| N | 13724 |
| Woman (N, %) | 13660 (99.5) |
| Age (years) | 38.19 (8.66) |
| Baseline weight (kg) | 58.50 (4.49) |
| Baseline BMI (kg/m^2^) | 22.98 (0.75) |
| Baseline PBF (%) | 31.29 (1.30) |
| Baseline SMR (%) | 40.05 (0.89) |
| Baseline FMR | 0.78 (0.06) |
| Baseline VAI | 5.73 (0.71) |
| Measurement frequency (times/year) | 51.51 (87.12) |

Continuous variables were described as mean (SD), “Measurement frequency” was described

as median (IQR), and categorical variables were described as count (percentage).
